# Leading Enterovirus Genotypes Causing Hand, Foot, and Mouth Disease in Guangzhou, China: Relationship with Climate and Vaccination against EV71

**DOI:** 10.3390/ijerph18010292

**Published:** 2021-01-02

**Authors:** Zhicheng Du, Yong Huang, Wayne R. Lawrence, Jianxiong Xu, Zhicong Yang, Jianyun Lu, Zhoubin Zhang, Yuantao Hao

**Affiliations:** 1 Department of Medical Statistics, School of Public Health, Sun Yat-sen University, Guangzhou 510080, China; duzhch5@mail.sysu.edu.cn; 2 Department of Immunization Programme Planning, Guangzhou Center for Disease Control and Prevention, Guangzhou 510440, China; gzcdc_huangy@gz.gov.cn (Y.H.); gzcdc_xujx@gz.gov.cn (J.X.); 3 Department of Epidemiology and Biostatistics, School of Public Health, State University of New York at Albany, Albany, NY 12144, USA; wlawrence@albany.edu; 4 Department of Communicable Disease Control and Prevention, Guangzhou Center for Disease Control and Prevention, Guangzhou 510440, China; gzcdc_yangzhc@gz.gov.cn (Z.Y.); gzcdc_lujy@gz.gov.cn (J.L.)

**Keywords:** hand, foot, and mouth disease, genotype frequency, climate

## Abstract

(1) Background: Assignment of pathogens to the correct genus, species, and type is vital for controlling infectious epidemics. However, the role of different enteroviruses during hand, foot, and mouth disease (HFMD) epidemics and the major contributing factors remain unknown. (2) Methods: HFMD cases from 2016 to 2018 in Guangzhou, China were collected. The relationship between HFMD cases and genotype frequency, as well as the association between genotype frequency and climate factors, were studied using general linear models. We transformed the genotype frequency to the isometric log-ratio (ILR) components included in the model. Additionally, vaccination rates were adjusted in the climate-driven models. (3) Results: We observed seasonal trends in HFMD cases, genotype frequency, and climate factors. The model regressing case numbers on genotype frequency revealed negative associations with both the ILRs of CAV16 (RR = 0.725, *p* < 0.001) and EV71 (RR = 0.421, *p* < 0.001). The model regressing genotype frequency on driven factors showed that the trends for EV71 proportions were inversely related to vaccination rate (%, β = −0.152, *p* = 0.098) and temperature (°C, β = −0.065, *p* = 0.004). Additionally, the trends for CVA16 proportions were inversely related to vaccination rate (%, β = −0.461, *p* = 0.004) and temperature (°C, β = −0.068, *p* = 0.031). The overall trends for genotype frequency showed that EV71 decreased significantly, while the trends for CVA16 increased annually. (4) Conclusions: Our findings suggest a potential pathway for climate factors, genotype frequency, and HFMD cases. Our study is practical and useful for targeted prevention and control, and provides environmental-based evidence.

## 1. Introduction

Hand, foot, and mouth disease (HFMD) is a common and potentially fatal infectious disease in children under 5 years of age [1]. Deaths from HFMD occur infrequently [2], with an annual average of 191 deaths over the past five years in China [3]. In China, HFMD has caused substantial disease burden, and has remained at the top of the list for Group C infectious diseases in terms of morbidity and mortality since the May 2008 outbreak in Anhui [3]. In preschools, the presence of HFMD cases can lead to suspension of classes [4]. The parents/guardians of infected children are often financially impacted due to income lost resulting from not working in order to care for their child. Therefore, HFMD remains a major societal concern.

Variation in genotype frequency is potentially an important contributor for the consistently high number of HFMD cases in China. A few genotypes of enteroviruses including coxsackie virus A16 (CAV16), enterovirus 71 (EV71), CAV4, 5, 9, and 10, and CBV 2 and 5 are potentially the cause of HFMD [5]. In this study, genotype frequency was defined as the proportions of the enterovirus genotypes (EV71, CVA16, or Other enterovirus) that are responsible for causing HFMD. The pathogens that cause HFMD are RNA viruses that have a high mutation rate [6]. The EV71 monovalent vaccine, which was launched in 2016 [7], does not appear to have made a significant impact on the overall HFMD epidemic, potentially due to herd immunity not being achieved yet [8]. In addition, different enterovirus genotypes have dissimilar activity and transmission characteristics, which can cause cases of infection on differing scales [9]. Therefore, it is important to identify the enterovirus genotypes causing HFMD for prevention and control.

Climate factors may affect the HFMD epidemic by influencing the proportion of pathogens. The relationship between HFMD and temperature or relative humidity have been widely reported in previous studies [10,11,12]. The 91st percentile of temperature was estimated to have a risk ratio of 1.30 (95% CI: 1.23, 1.37) for HFMD incidence compared to the median level [11]. The attributable HFMD cases for temperature and relative humidity were reported as 815,942 (95% CI: 796,361–835,888) and 291,759 (95% CI: 226,183–358,494) [10]. However, the association between climate factors and HFMD genotype frequency rather than incidence remains not well understood.

This study will examine the relationship between HFMD cases and genotype frequency. We also investigated the association between HFMD genotype frequency and climate factors to develop a greater understanding on the potential mechanism of climate factors, genotype frequency, and HFMD cases.

## 2. Materials and Methods

### 2.1. Study Area

**Figure 1:**
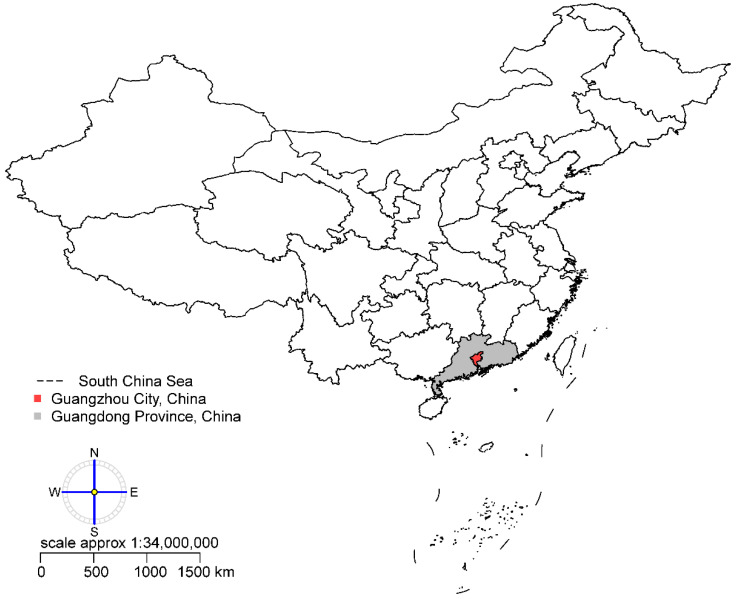
Geographic location of study area in Guangzhou, China.

The present study area was the city of Guangzhou (see Figure 1), one of China’s largest cities, and the capital of Guangdong province located in South China with a population of 14,904,400 in 2018 [13]. The area of Guangzhou is 7434.4 km^2^ on both sides from 112°57′ to 114°03′ E longitude and 22°26′ to 23°56′ N latitude in south-central Guangdong [13]. The city of Guangzhou has a humid subtropical climate, with a short, mild, and relatively dry winter, while also having a long, hot, and very wet summer [13].

### 2.2. Data Collection

HFMD is a notifiable disease in China, therefore all probable and confirmed HFMD cases must be reported online to the surveillance system (China Information System for Disease Control and Prevention) within 24 h of diagnosis using a standardized form. We collected data on HFMD cases from the surveillance system managed by China Center for Disease Control (CDC) in Guangzhou from January 2016 to December 2018. The CDC collects data for all HFMD diagnoses by law, including date of birth, onset date, and enterovirus genotypes. Detailed information on genotype detection can be found from guidelines issued by the National Health Commission of China [14]. The enterovirus genotypes are determined using fluorescence quantitative rRT-PCR (real-time reverse transcriptase PCR) for clinical specimens. The clinical specimens, including nasopharyngeal swabs and anal swabs or feces, are collected from sampling cases and kept at −80 °C until examination for enterovirus by rRT-PCR. Viral RNA was extracted from the clinical specimens using the Viral RNA Mini Kit in accordance with the manufacturer’s instructions and was subjected to rRT-PCR. Analysis of real-time data was used for the classification of the virus as EV71, CVA16, or Other enterovirus. The sampling cases are the earliest 5 (at least) mild cases and all severe cases per month from each sentinel site. All HFMD cases in Guangzhou were included in the current analysis. Three monthly time-series for the proportions from three reported enterovirus genotypes were calculated as the genotype frequency of HFMD.

Vaccination data were obtained from the surveillance system managed by the Guangzhou CDC. It is important to note that there is currently only a vaccine against EV71. The CDC collects date of birth, vaccination date, and cumulative vaccine doses for all inoculations administered in Guangzhou. We computed the monthly vaccination rate by dividing monthly number of vaccinations by the population under 5 years of age in each year. HFMD genotype frequency and vaccination rate were linked by calendar month.

Climate data including air temperature (°C) and relative humidity (%) were collected from China Meteorological Data Service Center (CMDC, http://data.cma.cn/en). There are two national weather stations located in Guangzhou. Each station records the daily average air temperature (°C) and relative humidity (%). Daily data from two stations were aggregated into monthly average data for Guangzhou and were linked with HFMD genotype frequency by the calendar month.

### 2.3. Statistical Analysis

To further analyze the genotype frequency of HFMD (i.e., three compositional time-series), we conducted isometric log-ratio transform (ILR) [15] for genotype frequency to obtain the ILR components. The ILR components can be analyzed without the limitation of collinearity and restriction limited to 0–1. Setting the compositional time-series of other enteroviruses, CVA16 and EV71, as *p*_i_, i = 1, 2, 3, the ILR components in each month with other enteroviruses as the reference are as follows:

(1) $$ILR_{CVA16}=\sqrt{\frac{1}{2}}\ln\left[\frac{g\left(p_{1}\right)}{p_{2}}\right]$$

(2) $$ILR_{EV71}=\sqrt{\frac{2}{3}}\ln\left[\frac{g\left(p_{1}+p_{2}\right)}{p_{3}}\right]$$

where *g*(·) denotes the geometric mean function and *p*_1_, *p*_2_, *p*_3_ are the proportions of other enteroviruses, CVA16, and EV71, respectively. By default, if one of pi is equal to 0, all ILR components for this month will set to 0.

First, we described the number of cases and genotype frequency using the time-series plots. Second, we examined the association between case numbers and genotype frequency by regressing case numbers on the ILR components using Poisson regression. The relative risk (RR) of the ILR component was extracted. Third, the driven factors (e.g., vaccination rate, temperature, humidity) were described using the time-series plots. Fourth, we used the univariate and multivariate linear regression, regressing the ILR components on the driven factors to examine the association between genotype frequency and driven factors. To control for unmeasured time-varying confounding, we used the natural cubic splines of calendar time with 4 df (common multiple number for 2 epidemic peaks and 4 seasons) per year to remove the long-term trends and seasonality [11]. Finally, the fitted and forecasted genotype frequency were conducted to validate the internal and external accuracy of the climate-pathogen model. In the present study, all data management and statistical analyses were conducted using R version 4.0.2 (R Foundation for Statistical Computing, Vienna, Austria; https://www.r-project.org/).

## 3. Results

### 3.1. Characteristics of HFMD Cases

Table 1 shows the characteristics of HFMD cases by genotyped and undetected in Guangzhou, China from 2016 to 2018. A total of 185,838 HFMD cases were reported to the Guangzhou CDC surveillance system from January 2016 to December 2018. Most HFMD cases were diagnosed in children at one year of age (29.82%), in males (60.36%), and were mild cases (100.00%). A total of 5067 (2.73%) cases completed a rRT-PCR detection. Among the cases genotyped, children at one year of age (31.40%), males (61.26%), and mild cases (99.90%) had similar distributions as undetected cases.

### 3.2. Associations between HFMD Cases and Genotype Frequency

**Figure 2:**
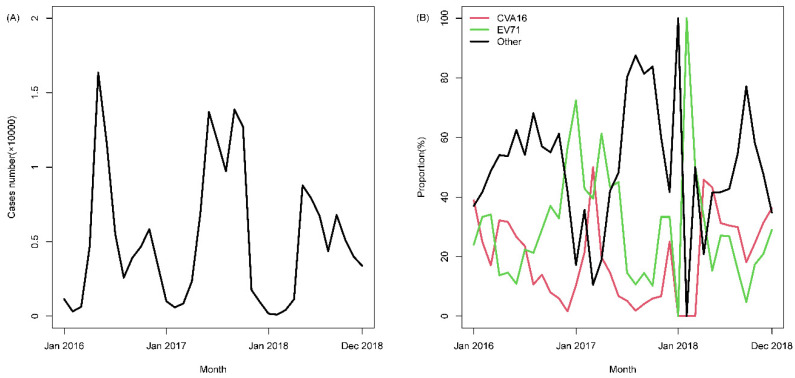
Observed trends in case numbers and genotype frequency of HFMD from 2016 to 2018 in Guangzhou, China. (**A**) Case number of HFMD; (**B**) genotype frequency of HFMD.

Figure 2 presents the seasonal trends in case numbers and genotype frequency for HFMD from 2016 to 2018 in Guangzhou, China. At the beginning of each calendar year (January), the case numbers continuously decreased to a low level, while the proportions of EV71 remained the predominant pathogen. During the middle of each calendar year (approximately April to October), the case numbers continuously reached a high level, while the proportion of other enteroviruses always stayed the predominant pathogen. The trends for CVA16 were comparable to those of EV71, with the proportions increasing annually. The results of regressing case numbers on genotype frequency shows that case numbers were negatively associated with both the ILRs of CAV16 (RR = 0.725, *p* < 0.001) and EV71 (RR = 0.421, *p* < 0.001).

### 3.3. Associations between Genotype Frequency and Climate Factors

**Figure 3:**
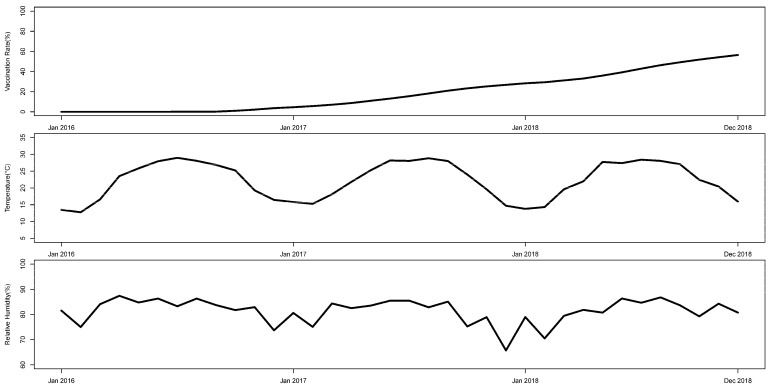
Observed trends in vaccination rates and climate factors from 2016 to 2018 in Guangzhou, China.

Figure 3 presents the time trends for vaccination rates and climate factors. We observed a crude relationship between these factors and case numbers, as well as genotype frequency for HFMD. Since the launch of the EV71 vaccine in 2016, the vaccination rate among children under 5 years of age increased slightly to approximately 50% at the end of 2018. Temperature had an expected seasonal trend that was analogous to the trends for HFMD case numbers. A positive relationship was observed between temperature and the proportions of other enteroviruses, while a negative relationship was observed for CVA16 and EV71. Similar findings were also observed for humidity.

Table 2 shows the association between HFMD genotype frequency and vaccination rate, as well as climate factors derived from univariate linear regressions. The vaccination rate (linear regression coefficient (β) = −0.235, *p* = 0.029), temperature (β = −0.062, *p* < 0.001), and humidity (β = −0.041, *p* = 0.040) were inversely related to trends for EV71 proportions. Similarly, the trends for CVA16 proportions were inversely related to vaccination rate (β = −0.562, *p* < 0.001) and temperature (β = −0.056, *p* = 0.024), while humidity (β = −0.009, *p* = 0.754) was not statistically significant.

Table 3 shows the association between HFMD genotype frequency and vaccination rate, as well as climate factors derived from the multivariate linear regression. The vaccination rate was inversely related to the trends for CVA16 (β = −0.461, *p* = 0.001) and EV71 (β = −0.152, *p* = 0.098) proportions. Temperature was inversely related to trends for CVA16 (β = −0.068, *p* = 0.031) and EV71 (β = −0.065, *p* = 0.004) proportions. However, humidity was not statistically associated with CVA16 (β = 0.041, *p* = 0.246) and EV71 (β = 0.011, *p* = 0.632) proportions. In Table 4, each unit increase in vaccination rate was associated with all changes for HFMD genotype frequency (13.84% increase for other enteroviruses, 8.77% increase for CVA16, and 5.07% decrease for EV71). Each unit increase in temperature was associated with all changes for HFMD genotype frequency (2.52% increase for other enteroviruses, 0.76% decrease for CVA16, and 1.76% decrease for EV71). Additionally, each unit increase in humidity was associated with all changes for HFMD genotype frequency (1.10% decrease for other enteroviruses, 0.80% increase for CVA16, and 0.30% increase for EV71).

**Figure 4:**
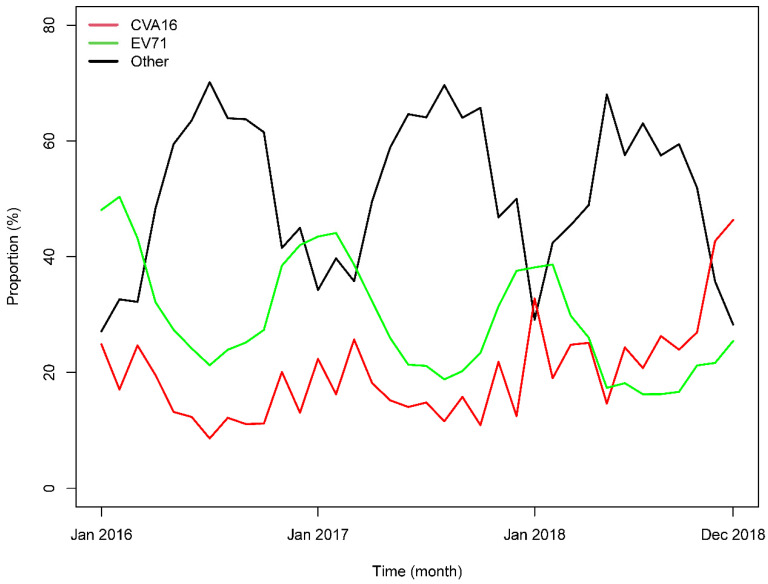
Fitted trends in genotype frequency of HFMD from 2016 to 2018 in Guangzhou, China.

Figure 4 presents fitted trends in genotype frequency for HFMD derived from the multivariate linear regression. The seasonality for the fitted trends was consistent with the observed trends. The trends for other enteroviruses decreased slightly, while EV71 decreased significantly. By contrast, the trends for CVA16 increased annually.

**Figure 5:**
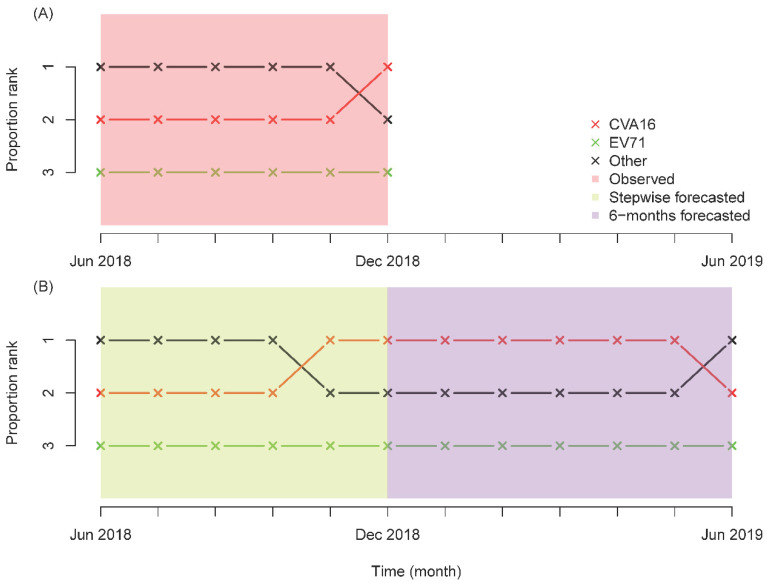
Predicted and observed trends in genotype frequency of HFMD in Guangzhou, China. (**A**) and (**B**) indicate the observed and forecasted genotype frequency, respectively.

Figure 5 presents the predicted performance and forecasted trends in genotype frequency for HFMD based on the multivariate linear regression. During the stepwise forecasting (June 2018 to December 2018), the result for only one month in November 2018 was incorrectly predicted. In the following six months, CVA16 became the predominant pathogen and then the secondary pathogen in June 2019. As expected, EV71 remained the lowest pathogen during the forecast period.

## 4. Discussion

Using a time-series design, this study examined the relationship between HFMD cases and enterovirus genotype frequency, as well as the association between genotype frequency and climate factors. Our findings revealed that trends for EV71 and other enteroviruses decreased annually while CVA16 increased. HFMD cases were negatively associated with ILR components for both ILR_CAV16_ and ILR_EV71_. Additionally, change in HFMD genotype frequency was associated with climate factors. Each unit increase in temperature resulted in a 0.76% and 1.76% decrease in CVA16 and EV71, respectively, while each unit increase in humidity caused 0.80% increase in CVA16 and 0.30% increase in EV71. To the best of our knowledge, this study is the first to present the role of genotype frequency in an endemic area.

Genotype frequency is an important factor to consider during a HFMD epidemic. The present study findings revealed a statistical association between HFMD cases and genotype frequency. The substantial cause of diseases is always due to genotypes sporadically emerging [16]. For instance, around the year 2015, CVA6 emerged as the main cause of HFMD worldwide [17]. CV-A16 was found as the predominant genotype that caused HFMD in Yantai City, Shandong Province, China from 2011–2015 [18]. Having a better understanding of genotype frequency would support the appropriate choice of genotypes in multivalent vaccines for targeting a disease. For example, we observed that the proportion of EV71 decreased annually [19]. Therefore, a multivalent vaccine besides the present EV71 vaccine is urgently needed [20].

Climate factors may affect the HFMD epidemic by influencing the proportion of pathogens. Previous studies have described the relationship between climate factors and HFMD in terms of viral activity and outdoor activities [12]. Findings in the present study are more biologically plausible due to showing that the trends for CVA16 proportions were inversely related to temperature and positively related to humidity. Therefore, we suggest a potential pathway for climate factors, genotype frequency, and HFMD cases.

The present EV71 vaccine is effective in prevention of HFMD. We found that the trends for CVA16 and EV71 proportions were inversely related to the vaccination rate. This finding is consistent with our previous studies assessing the effectiveness of the EV71 vaccine [8]. While an overall 1-year efficacy of monovalent EV71 vaccines of 95% has been reported, a bivalent EV71/CVA16 vaccine is potentially more cost effective [21]. For this reason, an expanded program for EV71 vaccination is immediately needed.

The models based on ILR transform are suitable for compositional data, such as genotype frequency. Applying ILR transform to compositional data has been validated in the scientific areas of medical imaging [22] and geosciences [23]. The genotype frequencies of a certain disease are classical compositional data. In addition, the ILR components can be used as both dependent and independent variables, providing great flexibility for its applications.

While this study offers several strengths, including the practical value of ILR transform and the potential pathway of climate factors, genotype frequency, and HFMD cases, there are several important limitations worth mentioning. First, this study is essentially ecological in nature and thus unable to rule out potential ecological fallacies. However, the associations we observed could prompt other studies of causal design. Second, cases were randomly sampled for laboratory confirmation/viral test but this was not performed for all cases. Nevertheless, this sampling information was representative of all HFMD cases due to similar distributions between genotyped and undetected cases. Our findings could be applied to settings with similar population contact patterns and climate conditions where the potential pathway of climate factors, genotype frequency, and HFMD cases might be similar. Third, the information of specific genotypes in other enteroviruses was not available in this study. The other enteroviruses may contain CVA6, CVA10, CVA12, CVA9, CVA2, CVA4, CVB2, CVB4, ECHO2, ECHO14, or ECHO18 [24,25]. Therefore, more enterovirus genotypes should be included in the surveillance system to improve the accuracy of predicting outbreak trends.

## 5. Conclusions

Our study contributes to the limited knowledge on quantifying the associations between HFMD cases and genotype frequency, as well as genotype frequency and climate factors. The trends for EV71 and other enteroviruses decreased annually, while CVA16 increased. HFMD cases were negatively associated with ILR components for both ILR_CAV16_ and ILR_EV71_. Each unit increase in temperature caused 0.76% and 1.76% decrease in CVA16 and EV71, respectively, while each unit increase in humidity caused 0.80% and 0.30% increase in CVA16 and EV71, respectively. Findings from this study reveal the role of genotype frequency in HFMD epidemics and suggest a potential pathway for climate factors, genotype frequency, and HFMD cases. This study can serve as a reference for other studies on the associations between infectious diseases and environmental factors. Moreover, our findings are useful for targeted prevention and control of HFMD.

## Figures and Tables

**Table 1:** Characteristics of HFMD (hand, foot, and mouth disease) cases by genotyped and undetected.

Variable	Total	Undetected	Genotyped
	*n*(%)	*n*(%)	*n*(%)
	185,838 (100)	180,771 (97.27)	5067 (2.73)
Year			
2016	60,685 (32.65)	58,815 (32.54)	1870 (36.91)
2017	76,195 (41.00)	74,117 (41.00)	2078 (41.01)
2018	48,958 (26.34)	47,839 (26.46)	1119 (22.08)
Age			
0+	22,449 (12.08)	21,745 (12.03)	704 (13.89)
1+	55,423 (29.82)	53,832 (29.78)	1591 (31.40)
2+	33,481 (18.02)	32,686 (18.08)	795 (15.69)
3+	33,288 (17.91)	32,368 (17.91)	920 (18.16)
4+	19,540 (10.51)	19,012 (10.52)	528 (10.42)
5+	9821 (5.28)	9535 (5.27)	286 (5.64)
6+	11,836 (6.37)	11,593 (6.41)	243 (4.80)
Sex			
Male	112,181 (60.36)	109,077 (60.34)	3104 (61.26)
Female	73,657 (39.64)	71,694 (39.66)	1963 (38.74)
Severity			
Mild cases	185,833 (100.00)	180,771 (100.00)	5062 (99.90)
Severe cases	5 (0.00)	0 (0.00)	5 (0.10)
Virus			
Undetected	180,771 (97.27)	180,771 (100.00)	—
CVA16	985 (0.53)	—	985 (19.44)
EV71	1202 (0.65)	—	1202 (23.72)
Other enterovirus	2880 (1.55)	—	2880 (56.84)

**Table 2:** Univariate linear regression of HFMD genotype frequency on vaccination rate and climate factors.

Factors	ILR_CVA16_		ILR_EV71_	
	β	95%CI	β	95%CI
Vaccination rate (%)	−0.562 *	(−0.809, −0.316)	−0.235 *	(−0.435, −0.035)
Temperature (°C)	−0.056 *	(−0.103, −0.010)	−0.062 *	(−0.088, −0.035)
Humidity (%)	−0.009	(−0.068, 0.049)	−0.041 *	(−0.078, −0.004)

Notes: ILR _CVA16_: the isometric log-ratio representing the proportion of coxsackievirus A16; ILR_EV71_: the isometric log-ratio representing the proportion of enterovirus 71. *: Statistical significance with *p* < 0.05.

**Table 3:** Multivariate linear regression of HFMD genotype frequency on vaccination rate and climate factors.

Factors	ILR_CVA16_		ILR_EV71_	
	β	95%CI	β	95%CI
Vaccination rate (%)	−0.461 *	(−0.713, −0.210)	−0.152	(−0.326, 0.022)
Temperature (°C)	−0.068 *	(−0.126, −0.009)	−0.065 *	(−0.105, −0.024)
Humidity (%)	0.041	(−0.027, 0.108)	0.011	(−0.035, 0.058)

Notes: ILR CVA16: the isometric log-ratio representing the proportion of coxsackievirus A16; ILREV71: the isometric log-ratio representing the proportion of enterovirus 71. *: statistical significance with *p* < 0.05.

**Table 4:** Proportions changed in HFMD genotype frequency with each unit increase in vaccination rate and climate factors.

Factors	Other Enteroviruses		CVA16		EV71	
	%	(*P*_2.5_, *P*_97.25_)	%	(*P*_2.5_, *P*_97.25_)	%	(*P*_2.5_, *P*_97.25_)
Vaccination rate (%)	13.84	(6.55, 20.38)	−8.77	(−13.86, −2.52)	−5.07	(−9.41, −0.30)
Temperature (°C)	2.52	(1.00, 3.99)	−0.76	(−2.20, 0.76)	−1.76	(−2.79, −0.71)
Humidity (%)	−1.10	(−2.76, 0.58)	0.80	(−0.92, 2.59)	0.30	(−1.03, 1.57)

Notes: CVA16: coxsackievirus A16; EV71: enterovirus 71.

## Data Availability

Data of HFMD cases and EV71 vaccination presented in this study are available on request from the Guangzhou Center for Disease Control and Prevention. The data are not publicly available due to data protection regulation. The climate data are openly available in China Meteorological Data Service Center (CMDC, http://data.cma.cn/en).
